# Supplementary material for: Optimization of Cellular Transduction by the HIV-Based Pseudovirus Platform with Pan-Coronavirus Spike Proteins
Source: Viruses. 2024 Sep 20;16(9):1492. doi: 10.3390/v16091492 (PMC11437443; doi:10.3390/v16091492)
Supplement: Supplementary file 1 [file viruses-16-01492-s001.zip › viruses-3172845-supplementary.pdf]

Supplemental data

Table S1. Effect of Pseudovirus Supernatant Collection Method on Transduction Efficiency

Variant	48 h<br>Unfiltered (RLU)/<br>Filtered (RLU)	Fold difference	72 h<br>Unfiltered (RLU)/<br>Filtered (RLU)	Fold difference
D614G	1931023/<br>1010789	1.91	71240/<br>69450	1.02
Beta	280452/<br>30739	9.13	52328/<br>9000	5.81
Delta	1579469/<br>1395085	1.13	400577/<br>142412	2.81
Omicron	238638/<br>113665	2.10	64107/<br>55905	1.14

Table S2. Timing of Infection -Transduction Efficiency

Variant	Infectivity (RLU)<br>Early Infection	Infectivity (RLU)<br>Late Infection	Fold difference
D614G	1956533	64051	30.5
Beta	299244	30251	9.89
Delta	2442948	45600	53.57
Omicron	480768	47059	10.21

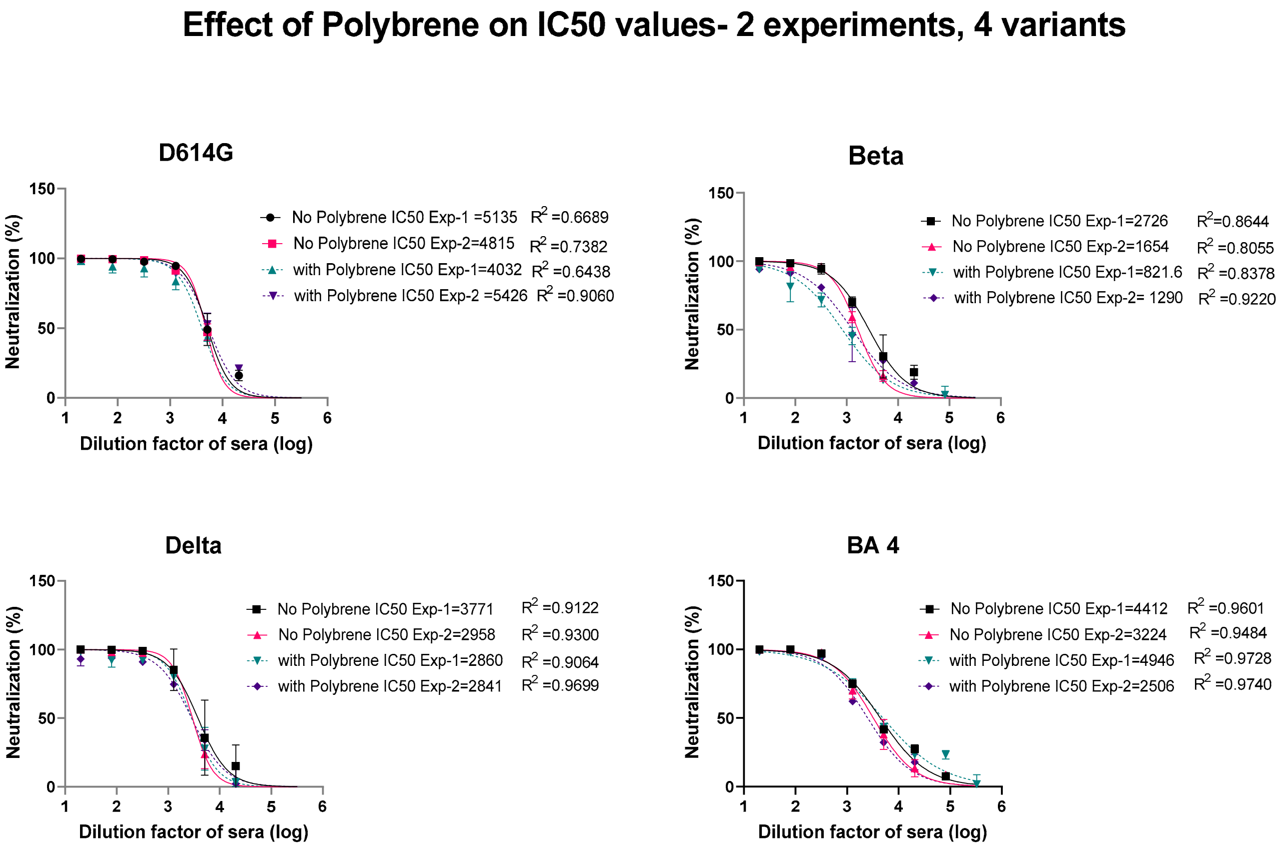

Figure S1. Effect of Polybrene on the improvement of transduction efficiency in neutralization assays. Polybrene did not negatively affect the neutralization reaction in at least three of the four variants, as observed by the alignment of the IC50 numbers and goodness of fit (R^2 values) in the presence vs. absence of polybrene. Slightly stringent IC50 numbers were observed in both experiments with the use of polybrene for neutralization of Beta variant as depicted by the neutralization curves.
